# Predictive Biomarkers of Acute Kidney Injury in COVID-19: Distinct Inflammatory Pathways in Patients with and Without Pre-Existing Chronic Kidney Disease

**DOI:** 10.3390/life15050720

**Published:** 2025-04-29

**Authors:** Caterina Carollo, Alida Benfante, Alessandra Sorce, Katia Montalbano, Emanuele Cirafici, Leonardo Calandra, Giulio Geraci, Giuseppe Mulè, Nicola Scichilone

**Affiliations:** 1 Unit of Nephrology and Dialysis, Hypertension Excellence Centre, Department of Health Promotion, Mother and Child Care, Internal Medicine and Medical Specialties (PROMISE), University of Palermo, 90133 Palermo, Italy; alessandra.sorce@community.unipa.it (A.S.); emanuele.cirafici@community.unipa.it (E.C.); leonardo.calandra@unipa.it (L.C.); giuseppe.mule@unipa.it (G.M.); 2 Department of Health Promotion Sciences, Maternal and Infant Care, Internal Medicine and Medical Specialties (PROMISE), University of Palermo, 90133 Palermo, Italy; alida.benfante@unipa.it (A.B.); nicola.scichilone@unipa.it (N.S.); 3 Nephrology and Dialysis Unit, Azienda Ospedaliera Ospedali Riuniti Villa Sofia Cervello, 90146 Palermo, Italy; 4 Department of Medicine and Surgery, “Kore” University of Enna, 94100 Enna, Italy; giulio.geraci@unikore.it

**Keywords:** SARS-CoV2, COVID-19, AKI, CKD, inflammation, NLR, IL-6

## Abstract

Background: Acute kidney injury (AKI) has emerged as a significant complication in patients with coronavirus disease 2019 (COVID-19). The pathophysiology of COVID-19-associated AKI is multifactorial, involving both direct viral effects on renal cells and indirect mechanisms such as systemic inflammation and cytokine storms. This highlights the critical need for early detection and effective management strategies to mitigate kidney injury and improve patient outcomes. The aim of our study is to assess the potential predictive role of inflammatory biomarkers in determining the risk of developing COVID-19-associated AKI in patients with and without pre-existing CKD. Methods: This study included 84 patients stratified by pre-existing chronic kidney disease (CKD) status. Demographic, clinical, and laboratory data were collected, including vital signs, hematological profiles, renal function markers, inflammatory biomarkers, coagulation parameters, and treatments. Outcomes such as acute kidney injury (AKI) and in-hospital mortality were documented. Results: In patients with pre-existing CKD, IL-6 and NLR demonstrated high predictive accuracy for AKI onset. In patients without pre-existing CKD, white blood cell (WBC) count emerged as a significant predictor of AKI onset. Conclusions: The differential roles of IL-6, NLR, and WBC in predicting AKI onset highlight distinct physiopathological pathways influenced by COVID-19. In CKD+ patients, chronic inflammation and immune dysregulation are key drivers of AKI, with IL-6 and NLR serving as robust markers of this inflammatory state. In contrast, in CKD− patients, AKI may be more influenced by acute inflammatory responses and infectious factors, as reflected by WBC count.

## 1. Introduction

Coronavirus disease 2019 (COVID-19), caused by severe acute respiratory syndrome coronavirus 2 (SARS-CoV-2), has precipitated a global health crisis since its emergence in late 2019 [1]. Beyond its primary respiratory manifestations, COVID-19 has been implicated in a spectrum of systemic complications, notably acute kidney injury (AKI) [2,3]. The incidence of AKI among hospitalized COVID-19 patients has varied widely, with studies reporting rates ranging from 20% to 40%, particularly among those requiring intensive care unit (ICU) admission [4]. A more recent comprehensive analysis of the medical literature, based on 27 studies encompassing a total of 502,593 individuals, has reported an overall AKI incidence of 31.8% (ranging from 0.5% to 56.9%) [5].

The wide variability in reported AKI incidence during COVID-19 infection can be attributed to differences in the diagnostic criteria used—such as KDIGO versus AKIN—as well as variations in patient populations across studies, particularly between critically ill individuals in intensive care units (ICUs), who tend to have higher rates of AKI, and those in general medical wards.

The development of AKI in the context of COVID-19 has been associated with increased morbidity and mortality, underscoring the need for early identification and intervention [6,7].

COVID-19 exhibits a broad clinical spectrum, with multiorgan involvement in infected patients. This is partially attributed to the widespread expression of angiotensin-converting enzyme 2 (ACE2), the molecular receptor that facilitates SARS-CoV-2 entry into host cells [8]. Renal impairment is frequently observed in COVID-19 patients, and SARS-CoV-2 affects the kidneys through multiple mechanisms.

During SARS-CoV-2 infection, the viral spike proteins bind to ACE2 receptors on the host cell surface and undergo cleavage by membrane proteases, enabling viral fusion and cellular entry. Renal cells are directly susceptible to infection due to ACE2 expression, which has been identified on the apical brush borders of proximal tubular cells, as well as on parietal epithelial cells, mesangial cells, and podocytes [9].

Beyond direct viral invasion, kidneys may also sustain damage through indirect pathways, including ischemia, coagulation abnormalities, multiorgan dysfunction, and immune dysregulation [10]. A hyperinflammatory response, commonly referred to as a “cytokine storm”, has been implicated in severe cases. This phenomenon is characterized by excessive and uncontrolled immune activation, which has also been observed in other critical conditions such as sepsis, malignancies, and severe viral infections [11].

It has been proposed that once ACE2 receptors are engaged by SARS-CoV-2, angiotensin II levels rise, leading to activation of angiotensin type 1 receptors (AT1Rs) and reduced angiotensin synthesis. This cascade is believed to contribute to the cytokine storm, which has been widely reported in critically ill COVID-19 patients. An overwhelming systemic inflammatory response can precipitate AKI, particularly in those with severe disease [12].

Higher rates of AKI have been documented in patients with severe COVID-19, especially those requiring intensive care. These patients have exhibited elevated levels of key inflammatory mediators, including interleukin-6 (IL-6), interleukin-8 (IL-8), tumor necrosis factor-alpha (TNF-α), interleukin-1 beta (IL-1β), and interferon-gamma (IFN-γ), compared to those with milder disease. This suggests a central role of the cytokine storm, in which proinflammatory cytokines such as IL-6, TNF-α, and IFN-γ are the primary drivers. The ensuing inflammatory cascade within the kidneys leads to increased vascular permeability, intrarenal inflammation, and volume depletion, ultimately resulting in AKI. This inflammatory milieu is believed to play a pivotal role in the onset and progression of AKI in COVID-19 patients [13].

In individuals without pre-existing chronic kidney disease (CKD), COVID-AKI often manifests abruptly, with varying degrees of severity. The recovery trajectory in these patients is heterogeneous; while some regain baseline renal function, others may experience persistent impairment, highlighting the unpredictable nature of renal recovery post-AKI. Conversely, patients with underlying CKD represent a particularly vulnerable cohort. The presence of CKD not only predisposes individuals to a higher risk of developing AKI during COVID-19 infection but also complicates the recovery process [14].

Studies have demonstrated that COVID-19 patients with pre-existing CKD who develop AKI are at an elevated risk for progression to end-stage kidney disease (ESKD) and have higher mortality rates compared to those without CKD [15,16]. This underscores the critical need for vigilant monitoring and tailored therapeutic strategies in this subgroup.

Inflammatory biomarkers have emerged as potential tools for predicting the development and severity of COVID-19 infection [17,18]. Markers such as C-reactive protein (CRP), procalcitonin, ferritin, lactate dehydrogenase (LDH), interleukin-6 (IL-6), and tumor necrosis factor-alpha (TNF-α) have been investigated for their prognostic utility. For instance, the neutrophil-to-lymphocyte ratio (NLR) has been identified as a simple yet effective marker for assessing inflammation and predicting AKI development in this context [19].

Despite these insights, specific data linking these biomarkers directly to adverse renal outcomes such as AKI remain limited, and the predictive role of inflammatory biomarkers in the development of COVID-AKI, particularly in patients with and without pre-existing CKD, has not yet been fully elucidated. Further research is warranted to clarify the mechanisms linking systemic inflammation to renal injury in COVID-19 and to validate the clinical utility of these biomarkers in diverse patient populations.

The aim of our study is to assess the potential predictive role of inflammatory biomarkers in determining the risk of developing COVID-AKI in patients with and without pre-existing CKD.

## 2. Materials and Methods

### 2.1. Study Design and Population

We conducted a retrospective observational study on a cohort of patients diagnosed with coronavirus disease 2019 (COVID-19) who were consecutively admitted to the inpatient unit of the Pulmonology Department at the Policlinico of Palermo during the first pandemic wave. A total of 90 patients were consecutively enrolled, of whom 84 met the exclusion criteria detailed in the study design below:

- No past or present medical history of autoimmune diseases, malignancies, or inflammatory chronic diseases;
- Patients with a history of kidney transplantation and/or with acute kidney injury prior to hospital admission;
- Patients receiving nephrotoxic medications before hospitalization that could confound AKI development;
- Patients with insufficient clinical or laboratory data to assess renal function at admission and during hospitalization.

The study was conducted in accordance with the Declaration of Helsinki, and ethical approval was obtained from the institutional review board.

### 2.2. Patient Stratification

The study population was stratified into two groups based on the presence or absence of pre-existing chronic kidney disease (CKD) at the time of hospital admission. CKD was defined according to the Kidney Disease: Improving Global Outcomes (KDIGO) criteria, classifying patients with an eGFR < 60 mL/min/1.73 m^2^ at admission as having CKD [20].

Each of these two primary groups was further subdivided based on the development of AKI during hospitalization, as defined by the KDIGO consensus criteria for AKI [21], which include any of the following:

- An increase in serum creatinine (SCr) by ≥0.3 mg/dL within 48 h;
- An increase in SCr to ≥1.5 times baseline within seven days;
- A urine output reduction to <0.5 mL/kg/h for more than six hours.

### 2.3. Data Collection and Clinical Parameters

**Figure 1:**
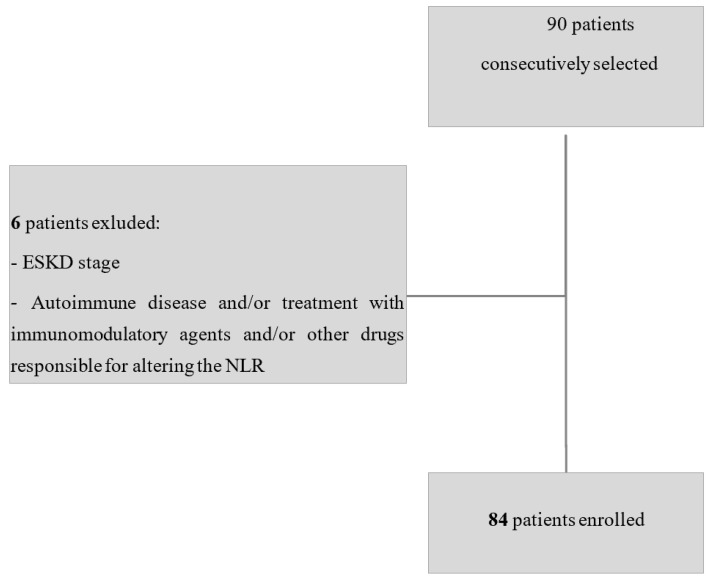
Flow chart of patient selection.

Demographic, clinical, and laboratory data were collected from electronic medical records at admission and throughout hospitalization. Patient demographics included age and sex, while medical history encompassed past comorbidities and chronic medication use. Vital signs recorded at admission included blood pressure and heart rate. Laboratory parameters were assessed at baseline and monitored during hospitalization, including hematological profiles such as complete blood count, renal function markers, including serum creatinine and estimated glomerular filtration rate (eGFR) calculated using the Chronic Kidney Disease Epidemiology Collaboration (CKD-EPI) equation, and inflammatory biomarkers such as interleukin-6 (IL-6), C-reactive protein (CRP), procalcitonin (PCT), and ferritin. The neutrophil-to-lymphocyte ratio (NLR) was determined by dividing the absolute neutrophil count by the absolute lymphocyte count (NLR = neutrophils/lymphocytes). Coagulation parameters, including D-dimer, fibrinogen, prothrombin time, and activated partial thromboplastin time, were also recorded. Additionally, data regarding medical treatments administered during hospitalization, including antiviral agents, corticosteroids, anticoagulants, and other supportive therapies, were extracted. Clinical outcomes, including the occurrence of AKI and in-hospital mortality, were documented from medical records and hospital discharge summaries. Prior to conducting statistical analyses, relevant data were processed and organized to ensure accuracy and clarity in presentation. Figure 1 shows the flow chart of patient selection.

### 2.4. Statistical Analysis

Statistical analyses were conducted using JASP 0.15 (University of Amsterdam, Amsterdam, The Netherlands) and IBM-SPSS package version 26 (IBM Corporation, New York, NY, USA). Categorical variables were summarized as absolute frequencies and corresponding percentages. The distribution of continuous variables was assessed using the Kolmogorov–Smirnov test. Variables exhibiting a normal distribution were reported as mean ± standard deviation (SD), while non-normally distributed variables were presented as median with interquartile range (IQR).

The study cohort was stratified into two groups based on the presence or absence of pre-existing chronic kidney disease (CKD). Between-group comparisons for categorical variables were conducted using the chi-square test or Fisher’s exact test, as appropriate. For continuous variables, one-way analysis of variance (ANOVA) was utilized when the assumption of normality was met, whereas the Kruskal–Wallis test was applied for variables that did not satisfy normality assumptions.

Correlation analyses between inflammatory markers and selected clinical or laboratory parameters were carried out using non-parametric methods, specifically Spearman’s rank correlation coefficient or Kendall’s tau, depending on the distributional characteristics of the variables involved.

Receiver operating characteristic (ROC) curve analysis was employed to determine optimal threshold values for the biomarkers under investigation. Sensitivity, specificity, and the area under the curve (AUC) were calculated to evaluate their discriminative ability for the prediction of the primary outcome.

The association between inflammatory biomarkers and the incidence of acute kidney injury (AKI) was initially assessed via univariate analyses and further evaluated using time-to-event methods. Kaplan–Meier survival curves were generated, and the log-rank test was applied to assess differences between groups. Subsequently, multivariable Cox proportional hazards regression was performed, incorporating both conventional risk factors and variables demonstrating statistical significance in univariate analyses.

All hypothesis testing was two-sided, with a significance threshold set at *p* < 0.05.

## 3. Results

The clinical characteristics, biochemical parameters, comorbidities, and in-hospital treatments of the study population were analyzed, comparing patients with pre-existing chronic kidney disease (CKD+) to those without (CKD−). The overall cohort included 84 patients, of whom 25 (29.8%) had a known history of CKD (Table 1).

Patients in the CKD+ group were significantly older than those in the CKD− group (78 ± 11 vs. 59 ± 14 years, *p* < 0.0001). While there was a higher proportion of male patients in the CKD− group (78% vs. 63%), the difference was not statistically significant (*p* = 0.126). No significant differences were observed in systolic blood pressure between groups, but diastolic blood pressure and heart rate were both significantly lower in CKD+ patients (68 ± 8 vs. 76 ± 9 mmHg, *p* < 0.0001; and 78 ± 11 vs. 86 ± 15 bpm, *p* = 0.014, respectively).

Renal function parameters confirmed the expected impairment in the CKD+ group, with significantly higher baseline creatinine levels (1.46 vs. 0.79 mg/dL, *p* < 0.0001) and reduced estimated glomerular filtration rate (35.6 ± 13 vs. 92.6 ± 16 mL/min/1.73m^2^, *p* < 0.0001). Inflammatory markers were markedly elevated in CKD+ patients, including interleukin-6 (median 49.3 vs. 23.6 pg/mL, *p* = 0.003), C-reactive protein (85.3 vs. 48.1 mg/L, *p* = 0.05), neutrophil-to-lymphocyte ratio (9 vs. 5, *p* < 0.0001), procalcitonin (0.19 vs. 0.08 µg/L, *p* = 0.008), and D-dimer levels (1173 vs. 604 ng/mL, *p* = 0.006). Ferritin levels did not differ significantly between groups.

Hematological parameters showed significantly lower hemoglobin concentrations in CKD+ patients (12.6 vs. 14 g/dL, *p* = 0.023), as well as reduced albumin levels (30 vs. 35 g/L, *p* = 0.0002), while no differences were found in total white blood cell count, platelet count, or creatine kinase levels.

Regarding comorbidities, there was a higher prevalence of myocardial infarction, ischemic heart disease, and heart failure in the CKD+ group, although these differences did not reach statistical significance. Chronic obstructive pulmonary disease was significantly more common in CKD+ patients (12% vs. 1.7%, *p* = 0.043), as was the occurrence of acute kidney injury during hospitalization (32% vs. 11%, *p* = 0.028). No significant differences were observed in the prevalence of hypertension, diabetes mellitus, or cerebrovascular disease.

**Figure 2:**
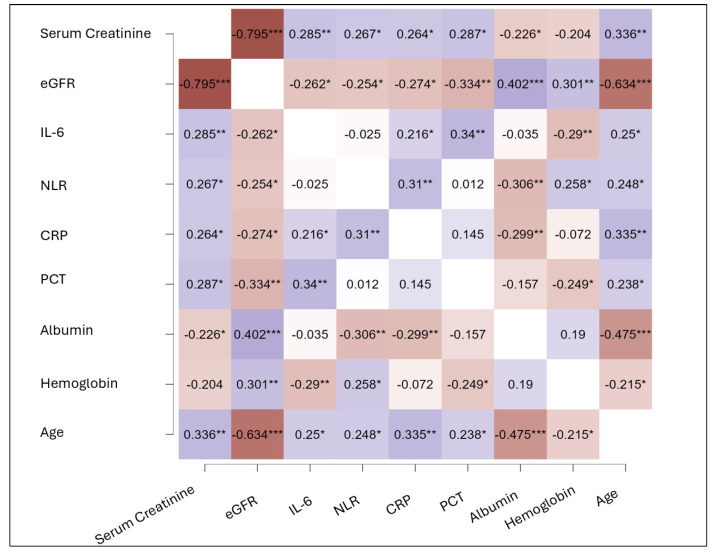
Correlation matrix of clinical and biochemical parameters. The heatmap displays correlation coefficients between variables related to kidney function, inflammation, and clinical characteristics. Positive correlations are shown in red, while negative correlations are represented in blue. The intensity of the color reflects the strength of the correlation. Statistically significant correlations are indicated as follows: *p* < 0.05 (*), *p* < 0.01 (**), *p* < 0.001 (***). Abbreviations: eGFR = estimated glomerular filtration rate; IL-6 = interleukin-6; NLR = neutrophil-to-lymphocyte ratio; CRP = C-reactive protein; PCT = procalcitonin.

The correlation analysis revealed significant associations between key clinical parameters related to kidney function, inflammation, and patient characteristics (Figure 2). Serum creatinine showed positive correlations with inflammatory markers, including interleukin-6 (IL-6, r = 0.285, *p* < 0.01), C-reactive protein (CRP, r = 0.264, *p* < 0.05), and procalcitonin (PCT, r = 0.287, *p* < 0.01).

Conversely, eGFR was negatively correlated with IL-6 (*r* = −0.262, *p* < 0.05), neutrophil-to-lymphocyte ratio (NLR, *r* = −0.254, *p* < 0.05), CRP (*r* = −0.274, *p* < 0.05), and, particularly, with PCT (*r* = −0.334, *p* < 0.01). Moreover, eGFR showed a strong positive correlation with albumin levels (*r* = 0.402, *p* < 0.001), indicating that patients with better kidney function also had improved nutritional and inflammatory status.

Inflammatory biomarkers displayed significant intercorrelations, with IL-6 positively associated with CRP (*r* = 0.216, *p* < 0.05) and PCT (*r* = 0.34, *p* < 0.01). NLR also correlated positively with CRP (*r* = 0.31, *p* < 0.01) and age (*r* = 0.248, *p* < 0.05), emphasizing its role as a marker of systemic inflammation, particularly in older patients.

Hemoglobin levels were inversely correlated with IL-6 (*r* = −0.29, *p* < 0.01), NLR (*r* = −0.306, *p* < 0.01), and PCT (*r* = −0.249, *p* < 0.05), further suggesting that systemic inflammation contributes to anemia. Similarly, albumin levels were inversely associated with inflammatory markers, including CRP (*r* = −0.299, *p* < 0.01) and NLR (*r* = −0.306, *p* < 0.01), while also showing a strong negative correlation with age (*r* = −0.475, *p* < 0.001), reflecting the combined impact of aging and inflammation on nutritional status.

**Figure 3:**
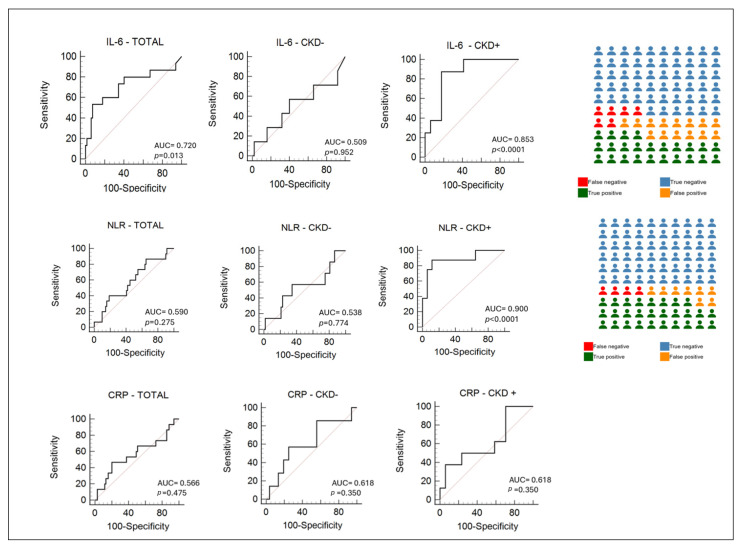
Diagnostic performance of IL-6, NLR, and CRP across total study cohort, patients with AKI without preexisting chronic kidney disease (CKD–), and those with preexisting CKD (CKD+). On the right side of the figure, icon plots are presented, visually representing the diagnostic classification performance of each marker specifically in the CKD+ population. True positives (green), true negatives (blue), false positives (orange), and false negatives (red) are depicted to illustrate the balance between sensitivity and specificity.

To assess the predictive value of inflammatory biomarkers for AKI onset, we first performed a receiver operating characteristic (ROC) curve analysis in both patients with preexisting CKD and those without prior renal impairment (Figure 3).

In the total population, IL-6 demonstrated moderate diagnostic accuracy, with an AUC of 0.720 (*p* = 0.013), suggesting a statistically significant ability to discriminate between patients with and without the condition of interest. NLR and CRP, on the other hand, showed low accuracy in this group, with AUCs of 0.590 (*p* = 0.275) and 0.566 (*p* = 0.475), respectively, indicating poor diagnostic performance.

Among CKD− patients, none of the three markers provided meaningful discrimination. IL-6 had an AUC of 0.509 (*p* = 0.952), NLR 0.538 (*p* = 0.774), and CRP 0.618 (*p* = 0.350). All results were statistically non-significant, suggesting that in patients, these markers are not reliable for diagnostic purposes.

Conversely, in the CKD+ subgroup, both IL-6 and NLR demonstrated high diagnostic accuracy, with IL-6 reaching an AUC of 0.853 (*p* < 0.0001) and NLR showing an excellent AUC of 0.900 (*p* < 0.0001). These findings suggest that IL-6 and NLR are particularly effective in identifying pathological states in patients with chronic kidney disease. At the optimal cut-off value of 6.4, determined using the Youden Index, the sensitivity and specificity are both 88%, reflecting a high capacity to correctly identify both positive and negative cases.

CRP, although slightly improved in this group compared to the others, remained a poor diagnostic tool, with an AUC of 0.618 (*p* = 0.350).

**Figure 4:**
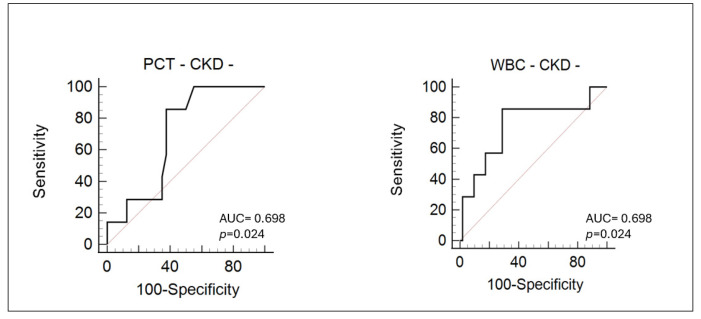
Diagnostic performance of Procalcitonin and white blood cell count in patients with AKI without preexisting chronic kidney disease.

The only predictive biomarkers for the onset of AKI in CKD− patients are Procalcitonin and total white blood cell count (Figure 4).

Both markers demonstrate moderate diagnostic performance, with identical AUC values of 0.698 and *p*-values of 0.024, indicating that their predictive capability is statistically significant in this specific patient subgroup. The AUC value, which approaches 0.7, suggests that PCT and WBC have acceptable discriminative power, though not outstanding, for identifying patients at risk of AKI in the absence of underlying chronic kidney impairment.

**Figure 5:**
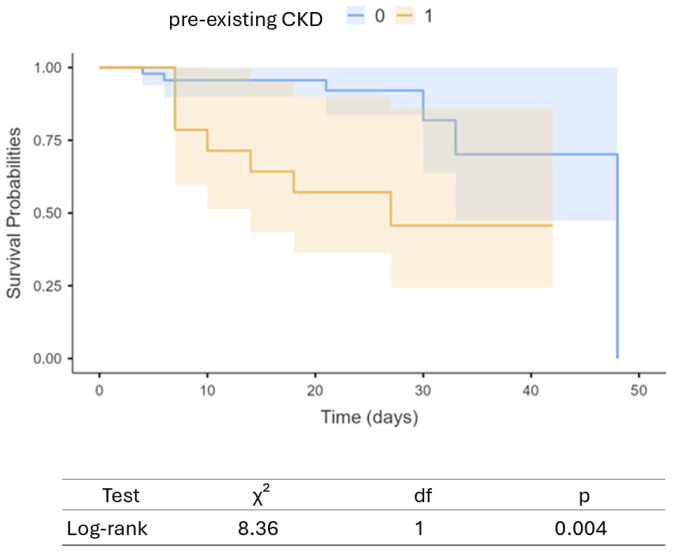
Kaplan–Meier survival curves comparing patients with (orange line) and without pre-existing chronic kidney disease (blue line). Shaded areas represent 95% confidence intervals.

We subsequently conducted an exploratory survival/time-to-event analysis using the Kaplan–Meier curve to compare patients belonging to the two different subgroups (Figure 5).

CKD− patients demonstrated significantly better survival probabilities over time compared to those with pre-existing CKD. The separation between the two survival curves becomes evident early in the follow-up period and persists throughout the observation window of 50 days. The statistical significance of this difference was assessed using the log-rank test, which yielded a chi-square (χ^2^) value of 8.36, resulting in a *p*-value of 0.004. This indicates a statistically significant difference in survival distributions between the two groups.

Finally, we adjusted the observed associations using Cox proportional hazards regression models to account for major confounding factors. A first model was conducted in CKD+, and a second model was applied to CKD− patients.

In the logistic regression model evaluating the occurrence of AKI in CKD+ patients, the only predictors retained were NLR (HR 1.04 95% C.I. 0.99–1.11, *p* = 0.001) and IL-6 (HR 1.02, 95% 1.01–1.02, *p* = 0.001). These markers were independently associated with the outcome, regardless of potential confounding factors such as sex, age, CRP, hemoglobin, eGFR, and comorbidities.

In the logistic regression model evaluating the occurrence of AKI in CKD− patients, the only predictor retained in the model was WBC (HR 1.0003, 95% C.I. 1.000–1.001, *p* = 0.021).

## 4. Discussion

AKI is a frequent and severe complication observed in patients with coronavirus disease 2019, particularly among those requiring hospitalization or intensive care. The incidence of COVID-19–AKI varies widely across studies, ranging from 5% to over 50% [5,22,23]. Methodological differences across studies—such as varying definitions of AKI, differences in study design, and the use of inconsistent diagnostic criteria—play a major role in shaping the reported outcomes. Additionally, patient characteristics, including age, pre-existing health conditions, severity of COVID-19 illness, and demographics, can significantly influence the likelihood of developing AKI, leading to variability between study populations. According to the literature, the incidence of AKI in the entire study population was 17.85%.

Importantly, the development of AKI in COVID-19 patients has been consistently associated with increased morbidity, prolonged hospital stays, higher rates of intensive care unit (ICU) admission, and significantly elevated mortality [24,25,26].

The pathophysiology of COVID-19–associated AKI is complex and multifactorial. Although it is well established that individuals with pre-existing health conditions are at greater risk of severe outcomes from SARS-CoV-2 infection, the exact mechanisms driving the virus’s pathogenicity remain only partially understood. Nevertheless, significant research has begun to illuminate both direct and indirect pathways by which COVID-19 may lead to AKI.

One hypothesized mechanism underlying COVID-19-AKI is the direct viral invasion of renal tissue by SARS-CoV-2. This process is thought to occur via the binding of the virus to ACE2-receptors, which are highly expressed on renal tubular epithelial cells and podocytes. The entry of the virus is further facilitated by the action of TMPRSS2, which primes the viral spike protein, allowing membrane fusion and cell entry. This ACE2/TMPRSS2-mediated pathway has been proposed as a central route by which SARS-CoV-2 could exert direct cytopathic effects on kidney cells, potentially contributing to tubular damage and glomerular dysfunction [27,28].

However, the evidence supporting this mechanism remains controversial. For example, Frithiof et al. reported a low detection rate of SARS-CoV-2 RNA in urine samples from critically ill patients, suggesting that active viral replication in the kidney may be uncommon or clinically insignificant in many cases [29]. Additionally, renal biopsy studies conducted in COVID-19 patients have provided inconsistent and inconclusive evidence of direct viral presence in kidney tissue [30,31]. While some autopsy studies initially described the presence of viral-like particles within renal cells via electron microscopy, subsequent investigations have cast doubt on these findings. Experts have raised the possibility that the observed structures may in fact represent normal cellular organelles, such as clathrin-coated vesicles or multivesicular bodies, rather than true viral particles [31,32].

These observations collectively imply that, although direct viral infection of the kidney is biologically plausible, it may not represent the predominant mechanism driving AKI in most COVID-19 patients. Instead, indirect pathways—such as systemic inflammation, endothelial injury, and microvascular thrombosis—are increasingly recognized as primary contributors to renal impairment in the setting of SARS-CoV-2 infection [33,34].

Immune dysregulation and cytokine storms, for example, can reduce lymphocyte counts, hinder viral clearance, and contribute to kidney dysfunction [35]. Other factors such as rhabdomyolysis—a condition characterized by muscle breakdown—can release harmful substances that damage the kidneys [36]. Additionally, inter-organ communication, particularly involving the lungs and heart, may exacerbate renal injury through systemic effects known as organ crosstalk [37].

One of the key drivers of organ damage in severe COVID-19 is the cytokine storm—an exaggerated systemic inflammatory response characterized by the release of high levels of proinflammatory cytokines such as IL-6, TNF-α, and IL-1β. This hyperinflammatory state contributes significantly to multi-organ dysfunction, including AKI. In this environment, various downstream molecular pathways are activated, among which the MMP-9/TIMP-1 axis plays a critical role [38].

It should be emphasized that, per se, individuals with CKD on conservative and hemodialysis treatment show, at baseline, a marked increase in MMP-9 [39].

In our study, we observed that among patients with pre-existing CKD, elevated NLR and IL-6 levels were independently associated with the development of AKI. Conversely, in patients without prior CKD, an increased white blood cell count was the sole independent predictor of AKI.

Elevated IL-6 levels have been consistently linked to renal function and susceptibility to ESRD [40] and to AKI in COVID-19 patients. Chen et al. demonstrated that higher serum IL-6 concentrations were significantly associated with renal impairment in COVID-19 cases, suggesting that IL-6 plays a pivotal role in the pathogenesis of AKI in this context [41]. Similarly, Filev et al. found that increased IL-6 levels were independently associated with mortality and AKI in COVID-19 patients with CKD [42]. These studies support our findings that IL-6 is a critical mediator of kidney injury in patients with underlying CKD.

NLR, a marker of systemic inflammation, reflects the balance between neutrophil-driven proinflammatory responses and lymphocyte-mediated immune regulation. In CKD+ patients, a high NLR indicates a shift towards a proinflammatory state, exacerbating renal injury through oxidative stress and immune dysregulation.

The neutrophil-to-lymphocyte ratio (NLR), a well-established marker of systemic inflammation, has also been implicated in the development of AKI [43,44]. An elevated NLR reflects an imbalance between neutrophils and lymphocytes, signaling an exaggerated inflammatory response. While specific studies directly correlating NLR with AKI in the context of COVID-19 are limited, increased NLR has consistently been associated with disease severity and poor clinical outcomes in COVID-19 patients, suggesting its value as a prognostic indicator [18,45].

Our findings reinforce the utility of NLR as a marker of inflammation-driven kidney injury, particularly in individuals with pre-existing CKD. In this subgroup, the NLR may be especially informative, as it reflects not only acute systemic inflammation but also the baseline state of chronic low-grade inflammation that is characteristic of CKD [46]. Notably, in CKD patients, an elevated NLR may result more prominently from lymphopenia—a condition that is already common in this population—rather than from neutrophilia [47]. This lymphocyte depletion is further exacerbated by the immunopathology of SARS-CoV-2 infection, which is known to induce profound lymphocyte apoptosis and impaired lymphopoiesis [48]. Moreover, the concurrent presence of neutrophilia may further contribute to lymphocyte suppression through indirect cytotoxic mechanisms [49]. Activated neutrophils can release inflammatory mediators and reactive oxygen species that impair lymphocyte survival and function, thereby amplifying immune dysregulation in severe cases of the disease.

As a result, the rise in NLR in CKD patients with COVID-19 may serve as a compounded signal of both chronic immune dysregulation and acute inflammatory stress, thereby offering a more nuanced reflection of the risk of AKI in this vulnerable group.

In CKD+ patients, NLR demonstrated not only high diagnostic accuracy for predicting AKI but may also reflect an increased burden of systemic inflammation associated with cardiovascular risk. Although the prevalence of myocardial infarction, ischemic heart disease, and heart failure was higher in the CKD+ group, these differences did not reach statistical significance, possibly due to limited sample size. However, the elevated NLR values observed in CKD+ patients (median 9.0 vs. 5.0 in CKD–, *p* < 0.0001) align with previous studies reporting its utility as a prognostic biomarker for cardiovascular events in CKD populations [50]. NLR has been proposed as an integrated marker of immune dysregulation and subclinical atherosclerosis, both of which are commonly exacerbated in the context of renal impairment. Thus, the elevated NLR in our CKD+ cohort may not only reflect inflammatory status related to kidney injury but also indicate heightened cardiovascular risk.

Interestingly, while several studies have reported a significant positive correlation between IL-6 and NLR in patients with COVID-19, particularly in severe or critically ill populations, we did not observe such an association in our cohort [51]. This discrepancy may be explained by several factors. First, IL-6 and NLR may follow different temporal kinetics: IL-6 is a rapidly inducible cytokine that peaks early during the cytokine storm, whereas NLR reflects a broader systemic inflammatory or stress response that may persist longer [18]. Second, IL-6 and NLR originate from different inflammatory axes—IL-6 being a cytokine produced by activated macrophages, endothelial cells, and fibroblasts, while NLR is shaped by bone marrow activity, neutrophil demargination, and lymphocyte apoptosis. These pathways may be differentially regulated, especially in patients with chronic comorbidities such as CKD, where baseline immune dysfunction could decouple typical inflammatory marker relationships. Finally, differences in patient severity compared to previous studies—ours potentially including a higher proportion of non-ICU or moderately ill cases—may have contributed to a less pronounced inflammatory response, resulting in weaker biomarker correlations.

In patients without prior CKD, our study identified elevated WBC count as the sole independent predictor of AKI. This observation is consistent with findings from other studies, where COVID-19 patients with AKI exhibited significantly higher WBC and neutrophil counts compared to those without AKI [52]. The elevated WBC count likely reflects an acute inflammatory response to SARS-CoV-2 infection, contributing to renal injury through mechanisms such as endothelial dysfunction, tubular damage, and microvascular thrombosis. Additionally, an increased WBC count may also signal the presence of secondary bacterial infections or superinfections, which are common complications in hospitalized patients with severe COVID-19, particularly those requiring intensive care or mechanical ventilation [53]. Superinfections further exacerbate systemic inflammation and immune dysregulation, placing additional stress on already vulnerable organs such as the kidneys. In this context, bacterial endotoxins and pathogen-associated molecular patterns (PAMPs) may amplify cytokine release and oxidative stress, intensifying renal inflammation and injury. Thus, leukocytosis in COVID-19 patients not only reflects the primary viral-induced inflammatory response but may also serve as an indicator of concurrent bacterial complications that contribute to the severity of AKI [54].

The differential predictors of AKI between patients with and without pre-existing CKD may be attributed to variations in baseline inflammatory states and immune responses. Patients with CKD often have a chronic proinflammatory milieu, rendering them more susceptible to additional inflammatory insults, as evidenced by the significant roles of IL-6 and NLR in this group. In contrast, individuals without prior CKD may experience a more acute inflammatory response, with WBC count serving as a marker of this reaction. This study is not without limitations. The retrospective design, the relatively small sample size, and furthermore, the absence of clinical severity scores and the lack of both internal and external validation, as well as a higher proportion of cases not in intensive care or those with moderate illness, restrict the generalizability of the findings. These limitations are partly attributable to the context in which the study was conducted—during the first wave of the COVID-19 pandemic—which placed extraordinary strain on the Italian healthcare system.

## 5. Conclusions

In conclusion, NLR and IL-6 represent important biomarkers for predicting AKI in COVID-19 patients, particularly in those with pre-existing CKD. These markers provide valuable insight into the inflammatory pathways driving renal injury and could be used to identify high-risk patients, allowing for timely intervention and improved management strategies. The accessibility, affordability, and predictive power of the NLR makes it an invaluable tool in clinical settings.

## Figures and Tables

**Table 1:** Clinical characteristics, biochemical parameters, main comorbidities, and treatment during hospitalization of the study population and the two subgroups.

	Total (n = 84)	CKD+ (n = 25)	CKD− (n = 59)	*p*
Age (years)	65 ± 16	78 ± 11	59 ± 14	<0.0001 +19 [12.6–15.4])
Male sex (%)	51	63	78	0.126
SBP (mmHg)	134 ± 19	132 ± 21	135 ± 17	0.518
DBP (mmHg)	73 ± 9	68 ± 8	76 ± 9	<0.0001 −8 [−12–4])
HR (bpm)	84 ± 14	78 ± 11	86 ± 15	0.014 −8 [−14–2]
Baseline creatinine (mg/dL)	0.89 (0.82–1.01)	1.46 (1.19–2.34)	0.79 (0.68–0.92)	<0.0001 0.67 [0.3–1]
Baseline eGFR (mL/min/1.73mq)	76.0 ± 30	35.6 ± 13	92.6 ± 16	<0.0001 −57 [−71–43]
Baseline IL-6 (pg/mL)	27.4 (19.08–37.87)	49.3 (14.2–110)	23.6 (6.1–44.1)	0.003 −26 [9–42]
Baseline CRP (mg/L)	58.0 (40.7–74.7)	85.3 (26.16–145.7)	48.1 (12.4–94.7)	0.05
NLR	5.6 (4.77–6.65)	9.0 (5.4–22)	5.0 (2.7–8)	<0.0001 4 [2–6]
PCT (µg/L)	0.10 (0.07–0.12)	0.19 (0.08–1.2)	0.08 (0.05–0.12)	0.008 0.11 [0.04–0.18]
Ferritin (ng/mL)	784 (620–955)	811 (235–1455)	767 (453–1148)	0.995
WBC (/µL)	7654 (6893–8713)	8440 (4960–11,305)	7560 (5280–10,032)	0.282
Hb (g/dL)	13.7 (12.8–14.1)	12.6 (10.7–14)	14 (12.7–14.9)	0.023 −1.4 [−2–0.4]
PLT (/µL)	221,500 (203,000–252,368)	235,000 (178,250–358,750)	221,000 (173,250–282,000)	0.481
D-dimer (ng/mL)	800 (604–1142)	1173 (874–1845)	604 (370–1309)	0.006 569 [37–1100]
Fibrinogen (mg/dL)	600 ± 195	673 ± 222	568 ± 175	0.024 105 [41–169]
CK (U/L)	71 (56–91)	66 (35–141)	74 (46–110)	0.808
Albumin (g/L)	34 (33–35)	30 (28–34)	35 (32–38)	0.0002 −5 [−7–3]
MI (%)	7.8	16	5	0.09
Hypertensive heart disease (%)	5.6	4	7	0.623
Ischemic heart disease (%)	11.1	20	8.5	0.136
Stroke/TIA (%)	3.3	8	1.7	0.155
Heart failure (%)	7.8	12	3.4	0.127
Arterial hypertension (%)	48.9	52	49	0.811
Diabetes mellitus (%)	35.6	40	35	0.702
COPD (%)	4.4	12	1.7	0.043
AKI (%)	18	32	11	0.028
Length of hospital stay (days)	19.36 ± 11.43	18 ± 12	20 ± 11	0.676
Exitus (%)	20	52	7	<0.0001
Parenteral nutrition during hospitalization (%)	35	41	59	0.09
Steroids during hospitalization (%)	97.61	96	98	0.526
Remdesivir (%)	44.04	20	54	0.004
ASA (%)	21	36	15	0.034
ACE-i (%)	23.81	28	22	0.557
ARBs (%)	26	28	25	0.806
Thiazide diuretics (%)	5	4	5	0.831
Loop diuretics (%)	31	68	15	<0.0001
Potassium-sparing diuretics (%)	6	8	5	0.606

## Data Availability

The data presented in this study are available on reasonable request from the corresponding author.
